# Transcriptional versus metabolic control of cell fitness during cell competition

**DOI:** 10.1016/j.semcancer.2019.05.010

**Published:** 2020-06

**Authors:** Katerina Lawlor, Salvador Pérez-Montero, Ana Lima, Tristan A. Rodríguez

**Affiliations:** National Heart and Lung Institute, Imperial College London, Hammersmith Hospital Campus, Du Cane Road, London W12 0NN, UK

**Keywords:** Cell competition, Cell fitness, Metabolism, Apoptosis, Growth regulation, P53, Myc, Hippo, NF-κB, Stat

## Abstract

The maintenance of tissue homeostasis and health relies on the efficient removal of damaged or otherwise suboptimal cells. One way this is achieved is through cell competition, a fitness quality control mechanism that eliminates cells that are less fit than their neighbours. Through this process, cell competition has been shown to play diverse roles in development and in the adult, including in homeostasis and tumour suppression. However, over the last few years it has also become apparent that certain oncogenic mutations can provide cells with a competitive advantage that promotes their expansion via the elimination of surrounding wild-type cells. Thus, understanding how this process is initiated and regulated will provide important insights with relevance to a number of different research areas. A key question in cell competition is what determines the competitive fitness of a cell. Here, we will review what is known about this question by focussing on two non-mutually exclusive possibilities; first, that the activity of a subset of transcription factors determines competitive fitness, and second, that the outcome of cell competition is determined by the relative cellular metabolic status.

## 1 General introduction

**Fig. 1:**
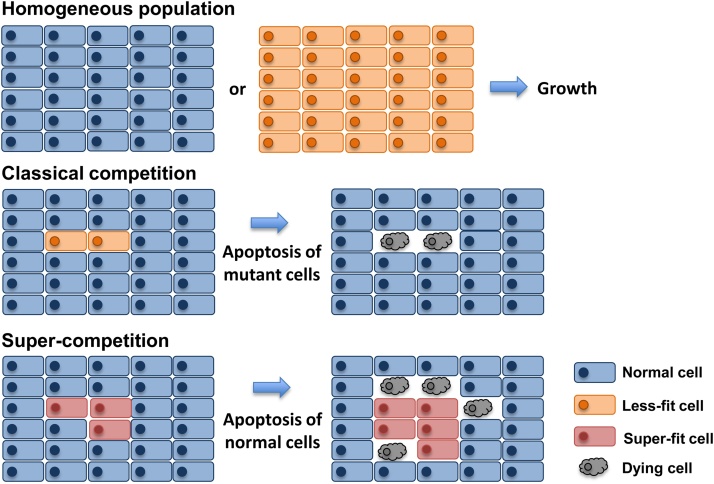
Competition versus Super-competition. Cell competition occurs between cells which are viable and proliferate in a heterogeneous environment. During competition cells which are defective or sub-optimal are eliminated when surrounded by wild-type cells. In contrast to this, during super-competition, cells which have acquired mutations that make them more competitive, induce apoptosis in the surrounding wild-type cells, highlighting the relative nature of cell competition.

In a multicellular organism, mechanisms to ensure the removal of damaged or suboptimal cells are essential for maintaining tissue health. It has been proposed that in addition to sensing their own intrinsic fitness, cells also determine their fitness relative to surrounding cells in the tissue. Through this mechanism, those cells that are comparatively weaker than their neighbours (generically called losers) can be eliminated, even though their defects are not sufficiently severe to trigger the intrinsic apoptotic pathway (summarised in Fig. 1). This process has been termed cell competition and accumulating evidence indicates that it has diverse functions in development, homeostasis and tumour suppression. However, in addition to its beneficial role in eliminating defective and potentially harmful cells, competition has also been implicated in cancer progression. This has been primarily observed through a process termed super-competition, by which cells acquire mutations that give them a competitive advantage and allow them to induce the elimination of surrounding wild-type cells. Given this behaviour, these mutant cells are generically termed super-competitors.

Much remains to be understood about the mechanisms by which cells are eliminated through competitive interactions. In particular, the factors which determine cell fitness and the process by which cells are able to recognise their relative fitness levels remain elusive. Here we will approach the first of these questions by contrasting what is known about the transcriptional versus the metabolic regulation of cell competition. For example, while a number of transcription factors have been shown to be critical for cell competition, most notably Myc, p53, NF-κB and TEAD, it is often unclear which of their transcriptional targets are acting downstream to regulate the outcome of competition. In some cases, these transcription factors may directly regulate pro- or anti-apoptotic genes, however in others, they appear to act further upstream in the process of competition to define the winner or loser status of a cell. One way in which these factors could affect cell fitness is through the regulation of metabolic pathways, as all these transcription factors have been shown to regulate genes involved in cell metabolism. Indeed, there is accumulating evidence that anabolic differences between neighbouring cells can affect cell fitness in competitive environments. This review will therefore seek to outline what is known about the transcriptional and metabolic regulation of cell competition and to explore the intersection between these two processes.

## 2 Transcriptional regulation of cell competition

The best-characterised transcriptional regulator of cell competition is Myc, overexpression of which can convert cells into super-competitors. Additionally, a variety of other factors with transcriptional activities, such as p53, NF-κB, TEAD/Scalloped and STAT, have all been shown to either affect the outcome of cell competition or to induce super-competition. Here we will describe what is known about the roles of each one of these factors in competition (summarised in Table 1).

**Table 1:** Transcription factors in cell competition. Summary of known transcription factors that play a role in several cell competition contexts as well the activities they produce. Dros. (*Drosophila*), Mam. (mammals).

**Transcription Factors**	**Competition context**	**Model**	**Activity**	**Key Studies**
**p53**	Irradiated bone marrow	Mam.	Increased p53 in losers	Bondar and Medzhitov [24]
	*Myc* overexpression in epithelial cells	Dros.	Increased p53 in winners	de la Cova et al. [28]
	*Bmpr1a^-/-^* and 4n mESCs	Mam.	Increased p53 in losers	Bowling [26]
	*Scrib^kD^* MDCK cells	Mam.	Increased p53 in losers	Wagstaff [27]
	*Mdm2^+/-^, Mdm4^+/-^* embryos	Mam.	Increased p53 in losers	Zhang et al. [25]

**Myc**	Myc overexpression in epithelial cells	Dros.	Increased Myc activity induces super-competition and increases p53 levels	de la Cova [4] Moreno and Basler[3] de la Cova et al. [28]
	*Bmpr1a^-/-^* or Tetraploid mESCs	Mam.	Decreased MYC in losers	Sancho et al. [15]
	*Scrib*^-^ epithelial cells	Dros.	Increased Myc activity rescues loser cell elimination	Chen et al. [9]
	*Lgl*^-^ epithelial cells	Dros.	Decreased Myc levels	Froldi et al. [14]
	*Myc* overexpression in mouse cardiomyocytes and epiblast cells	Mam.	High MYC levels produces super-competition	Clavería et al. [16] Villa del Campo et al. [17,18], Díaz-Díaz et al. [87]
	*MYC* inhibition in cancer cell lines	Mam.	Decreased MYC induces loser status	Di Giacomo et al. [21] Patel et al. [20]
	*Myc* overexpression in S2 cells	Dros.	Increased Myc induces winner status	Senoo-Matsuda and Johnston [19]

**NF-κB**	*Minute^+/-^*epithelial cells	Dros.	Increased activity in losers	Meyer et al. [54] Germani et al. [55]
	*Myc* overexpression in in epithelial cells	Dros.	Increased activity in losers	Meyer et al. [54] Alpar et al. [88] Germani et al. [55]
	*Scrib^-^* epithelial cells	Dros.	Increased activity in winners	Katsukawa et al. [56]

**STAT**	*Minute^+/-^*; *Mahjong^+/-^* epithelial cells	Dros.	STAT promotes winner proliferation	Kolahgar et al. [34] Kucinski et al. [35]
	*Stat92E^85C9^* epithelial cells and sustained STAT in epithelial cells	Dros.	Decreased STAT induces loser status and STAT activity induces super-competition	Rodrigues et al. [33]

**YAP/Yki** **TEAD/** **Scalloped**	*Scrib^-^* epithelial cells	Dros.	Suppressed Yki activity in losers	Chen et al. [9] Yamamoto et al. [39]
	*lgl^-^* epithelial cells	Dros.	Increased Yki activity in winners	Menéndez et al. [38]
	*Minute^+/-^* epithelial cells	Dros.	Increased Yki activity rescues loser elimination	Tyler et al. [40]
	*APC^-/-^* epithelial cells	Dros.	Increased Yki activity rescues loser elimination	Suijkerbuijk et al. [42]
	Hippo pathway manipulation in epithelial cells	Dros.	Increased Yki activity induces super-competition	Ziosi et al. [43]
	Hippo pathway manipulation in embryonic fibroblasts	Mam.	Increased Yki activity induces super-competition, decreased Yki activity induces loser status	Mamada et al. [44]
	Hippo pathway manipulation in the epiblast	Mam.	Decreased TEAD activity induces loser status	Hashimoto and Sasaki [45]

### 2.1 Myc

The proto-oncogene MYC is overexpressed in the vast majority of human cancers and thus has been the focus of intense research interest for some time. MYC transcription factors control a wide range of cellular processes, including cell growth, proliferation, ribosomal biogenesis, protein synthesis, glycolysis and mitochondrial biogenesis [1,2]. Moreover, MYC transcriptional activity is modulated in a context-dependent manner by a number of signalling pathways, including by the Hippo pathway, which, as we will discuss later, also regulates cell competition.

Myc was first demonstrated to play a role in competition through its ability to induce super-competition in the *Drosophila* wing imaginal disc. Here, epithelial cells with four copies of the *Myc* gene are able to trigger apoptosis in surrounding cells with normal Myc levels. Furthermore, once this elimination has taken place, the *Myc*-overexpressing cells grow to fill the space left by the dead cells without producing morphological abnormalities [3,4]. This phenotypically silent replacement of loser cells led to the hypothesis that cell competition regulates organ size, an attractive possibility that we have reviewed elsewhere [5].

From this initial role, the importance of Myc in cell competition rapidly expanded through a number of studies that analysed the mechanism of elimination of a range of defective cell types in the *Drosophila* wing imaginal disc, such as cells carrying *lethal giant larvae* (*lgl^−^*) and *scribble* (*scrib^−^*) gene mutations. Lgl and Scrib are scaffold proteins that bind to the lateral membrane and govern epithelial apical-basal polarity. Mutant flies for these polarity genes demonstrate neoplastic overgrowth that causes large tumourous masses (reviewed in [6]), however, when mutant cells are surrounded by wild-type tissue, they are eliminated by apoptosis [[7], [8], [9], [10], [11], [12], [13]]. *Lgl^−^* cells eliminated from wild-type imaginal discs express low *Myc* levels and *Myc* overexpression in *lgl^−^* or *scrib^−^* cells rescues their elimination [9,14]. In contrast to this, when *lgl^−^* cells are surrounded by cells carrying the *Minute* mutation, which makes them haploinsufficient for ribosomal proteins and have lower *Myc* expression, they are no longer eliminated [14]. Together, these results suggest that relative lower *Myc* levels is the cause of the elimination of these mutant cell types.

More recently, MYC has also been implicated in cell competition during mouse embryogenesis. In the early embryonic tissue, or epiblast, MYC expression is mosaic and those cells with low MYC levels die by apoptosis [15,16]. Similarly, embryonic stem cells that are eliminated by cell competition, such as those with defective BMP signalling, that are tetraploid or autophagy deficient, all display lower MYC expression than wild-type cells in co-culture [15]. Furthermore, in the epiblast and heart, *Myc* overexpression turns cells into super-competitors allowing them to replace wild-type cells [[16], [17], [18]]. Therefore, in mouse, just as in *Drosophila*, relative MYC levels determines the competitive ability of the cell.

Differences in MYC levels have also been suggested to induce cell competition in human cancer cells. The first demonstration that differences in MYC levels can also trigger cell competition between cell lines came in *Drosophila*, where S2 cells overexpressing Myc outcompete S2 cells that don’t [19]. More recently, in the human breast cancer line MCF7, shRNA inhibition of *MYC* was shown to lead to out-competition by control cells [20]. Furthermore, analysis of the tumour-stroma interface in a number of human tumour samples found a strong correlation between elevated MYC levels in the tumour and activated caspase-3 expression in the adjacent stroma [21]. This observation was supported by the finding that co-culturing human cancer cell lines with differing levels of MYC expression leads to increased apoptosis in those with lower MYC levels. Together, these results suggest that differences in MYC levels between tumour and stromal cells or within a tumour could aid tumour expansion by inducing super-competition.

From the above studies, it is clear that MYC is an important determinant of relative cell fitness, with winner cells having higher MYC levels than losers. However, in spite of these advances, the precise mechanism by which MYC affects cell fitness is not resolved. Given the importance of MYC in the regulation of cell anabolism, below we will review the different metabolic effects that MYC could have during cell competition.

### 2.2 P53

P53 is best known as a tumour suppressor that is mutated in about half of human tumours [22]. P53 was originally identified as a mediator of cell cycle arrest or apoptosis in the response to cellular stress. However, in recent years, it has become apparent that P53 can also regulate a plethora of other cellular processes including DNA repair, differentiation, stem cell reprogramming, metabolism and senescence amongst others (reviewed in [23]). The roles that p53 plays are usually complex and like Myc, they are also highly context dependent.

The first description for p53 in cell competition came in 2010, when the competitive behaviour of cells in the hematopoietic system was examined [24]. The authors used ionising radiation (IR) to induce cellular stress in hematopoietic stem and progenitor cells (HSPCs) and then compared their ability to repopulate the bone marrow of irradiated and non-irradiated mice. They observed that irradiated cells showed an increase in p53 expression and that this higher p53 expression caused them to be outcompeted by non-treated hematopoietic cells in the bone marrow. Importantly, the out-competition of the irradiated cells was found to be due to senescence rather than apoptosis [24]. A similar finding was made when mosaic mouse embryos and adult mice were analysed that are haploinsufficient for the *Mdm2* and *Mdm4* genes, two major negative regulators of p53 activity. Here the authors observed that mutation of these genes throughout the animal led to a moderate increase in p53 levels that had no effect on growth. In contrast to this, mosaic mutation of these genes provided the cells with a competitive disadvantage that was primarily thought to be due to a non-cell autonomous induction of growth arrest [25]. Also in mouse, defective cells eliminated by cell competition during early post-implantation stages show elevated p53 expression. In this context p53 is required for the repression of the mTOR pathway, an important metabolic regulator, in these defective cells specifically in a competitive environment and this repression induces apoptosis [26]. In Madin-Darby Canine Kidney (MDCK) cells, knockdown of *scribble* (*scrib^KD^*) in a sub-population of cells leads to their elimination by cell competition [11]. These cells also show elevated p53 expression, which makes them sensitive to tissue crowding. In a competitive environment with wild-type cells, this sensitivity causes mechanical stress and a further elevation of p53 levels that induces their elimination [27].

Differences in p53 levels during cell competition have also been reported in *Drosophila*. In Myc-induced competition, p53 is required in winner cells to sustain their metabolic and proliferation changes during competition and p53 mutation abolishes their ability to eliminate surrounding wild-type cells [28]. In contrast to this, apoptosis of *Minute* cells in mosaic imaginal discs was found to be independent of p53 levels as competition was still produced in p53 mutants [29]. Interestingly, *Minute* cells show elevated expression of the p53 target bZip-domain (*Xrp1*) during cell competition, which contributes to their reduced translation and cell growth rates and is essential for their competitive elimination [30]. However, this increase in *Xrp1* expression is also independent of p53 [31].

In summary, as is evident from the studies described above, the precise role of p53 in competition is highly context dependent, inducing growth arrest in some contexts and regulating apoptosis through the control of the metabolic state or mechanical properties of the cell in others.

### 2.3 STAT

The Janus kinase/signal transducers and activator of transcription (JAK-STAT) pathway is conserved from *Drosophila* to humans and is involved in multiple cellular processes including cell division, death, immunity and tumour formation. Activation of STATs occurs downstream of receptor-ligand (usually a cytokine) binding, which triggers JAK-mediated phosphorylation of tyrosine residues. This phosphorylation leads to dimerisation and translocation to the nucleus, where they can bind specific DNA sequences and activate transcription of target genes (reviewed in [32]).

A number of studies in *Drosophila* have shown that modulating JAK-STAT activity levels can affect competitive cell behaviour. In the wing and eye imaginal discs, wild-type cells induce surrounding cells with deficient JAK-STAT signalling to undergo apoptosis [33]. Conversely, cells with sustained JAK-STAT activation eliminate surrounding wild-type cells through Hid-dependent apoptosis [33]. In the *Drosophila* posterior midgut, *Minute* cells, both differentiated and stem cells, are eliminated by apoptosis when surrounded by wild-type cells. This is accompanied by increased stem cell proliferation and symmetric self-renewal of the wild-type cells [34]. During this process, chronic JNK activation in mutant cells induces expression of the JAK-STAT ligand Unpaired-3, which stimulates compensatory proliferation of the wild-type cells via JAK-STAT signalling. In contrast to this, in the imaginal wing disc, mutant cells for *Minute* and *Mahjong* (another polarity-associated gene) also display chronic JNK signalling, but this leads to increased JAK-STAT signalling that sustains their proliferation when all the cells in the fly are mutant, as well as promoting the proliferation of surrounding wild-type cells during cell competition [35].

The mechanism by which JAK-STAT signalling mediates cell competition is also still largely unknown. Although JAK-STAT is known to regulate *Myc* expression, JAK-STAT-induced cell competition appears to be largely independent of Myc, ribosomal biogenesis or other pathways involved in cell competition such as WNT/Wg, BMP/Dpp or Hippo signalling [33]. One intriguing possibility is raised by the fact that most of the JAK-STAT signalling ligands are cytokines (interferons or interleukins) involved in immunity and are regulated by STAT itself with NF-kB signalling cross-talk. Given the recent implication of NF-kB in cell competition that will be discussed below, it is possible that STAT signalling mediates cell competition by cooperating with NF-kB in the regulation of immune-like responses.

### 2.4 TEAD/Scalloped

TEADs are effectors of the Hippo pathway, a highly conserved kinase cascade involved in the cell-autonomous control of proliferation. Hippo (also known as MST1 and MST2 in humans) phosphorylates and activates large tumour suppressor 1/2 (LATS1/2) kinases, homologues of Warts in *Drosophila*, which then repress the activity of the transcriptional coactivators YAP and TAZ in mammals or their homologue Yorkie (Yki) in *Drosophila*. In mammals, the YAP/TAZ complex binds the TEAD family of transcription factors to activate genes involved in promoting proliferation and inhibiting apoptosis. In *Drosophila*, Yki interacts with the Scalloped transcription factor, which similarly drives the expression of pro-proliferative and pro-survival genes, such as *Cyclin E* and *Myc* [36,37]. Thus, repression of the Hippo pathway leads to increased YAP/TAZ or Yki activity and therefore, to increased proliferation and suppressed apoptosis via the regulation of TEAD/Scalloped activity.

Perhaps unsurprisingly, given its role in proliferation and apoptosis, numerous studies have implicated the Hippo pathway in cell competition. Indeed, increased Yki or YAP/TAZ activation is sufficient to rescue the elimination of a range of different loser cell types. For example, in the *Drosophila* imaginal wing disc, Yki activity is critical for the balance between elimination and overgrowth of polarity-deficient epithelial cells [9,38]. This is illustrated by the observation that Yki activation drives hyperproliferation and the formation of neoplastic masses when the whole tissue is composed of *scrib^−^* mutant cells, but if these *scrib^−^* cells are surrounded by wild-type cells, Yki activity is suppressed leading to their elimination [9]. Similarly, *lgl^−^* mutant cells partially evade elimination by competition if they express an oncogenic constitutively active form of Ras (Ras-V12), which increases Yki activity and therefore the proliferation rate of these double-mutant cells [38].

In the *Drosophila* adult eye, elimination of *scrib^−^* mutant clones by wild-type cells is mediated by the ligand Sas and its receptor PTP10D [39]. *Scrib^−^* cells in which PTP10D is knocked-down are not eliminated and demonstrate increased Yki nuclear localisation and expression of target genes. This increased Yki activity is critical for avoiding loser cell status, as in the absence of Sas-PTP10D signalling, JNK signalling switches from driving pro-apoptotic gene expression in loser cells to driving pro-proliferative gene expression through suppression of the Hippo pathway. Hippo pathway signalling has also been implicated in *Minute*-induced competition in the *Drosophila* eye. Here, increasing Yki activity in *Minute^-/+^* cells through mutation of negative regulators of the Hippo pathway, including Hippo itself, is sufficient to rescue them from elimination [40]. Similarly, wild-type cells with elevated Yki activity have an enhanced winner phenotype and eliminate *Minute^-/+^* cells more efficiently [41]. Moreover, adenomatous polyposis coli (APC)-null intestinal cells, which behave as super-competitors, also display increased Yki activity, and relative levels of Hippo pathway activity are crucial for determining the outcome of competitive interactions in this context [42]. Thus, the expression of Hippo pathway target genes appears to be a common feature in diverse competition processes.

The importance of Hippo pathway signalling in competition is further highlighted by the observation that elevated expression of Yki/YAP alone, or mutation of other members of the Hippo pathway, is sufficient to convert cells into super-competitors in both *Drosophila* [43] and mammalian models of competition [44]. Conversely, low Yki/YAP/TAZ activity is sufficient to confer loser status on cells [[44], [45], [46]]. Interestingly, it was recently demonstrated that human glioma cells that express lower levels of YAP are eliminated when surrounded by glioma cells with higher YAP expression [46]. The higher YAP-expressing cells in this system were also found to increase their proliferation and expression of tumourigenic genes specifically in this heterogeneous environment, indicating that these competitive interactions were enhancing tumourigenesis. However, the role of YAP activity in competition appears to be context-dependent as mammalian keratinocytes and MDCK cells expressing constitutively active YAP behave as losers in a competitive environment [47,48].

*Myc* is a transcriptional target of Yki/YAP/TAZ and thus one explanation for the crucial role of these transcription factors in competition is that they are upstream of Myc-induced competition ([43,49]). Indeed, in the study by Ziosi et al., the cell non-autonomous apoptosis induced by Yki overexpression was abrogated by *Myc* knock-down. In the pre-implantation mouse embryo, lower TEAD activity also triggers cell elimination by competition [45]. Here *Tead1^-/-^* cells show downregulated MYC expression, however reducing MYC activity throughout the embryo using a small molecule inhibitor did not rescue *Tead1^-/-^* cell elimination. This suggests that differences in MYC expression with wild-type cells is not the only reason for the elimination of these cells. In summary, although Myc appears to be an important target of TEAD/Scalloped factors during cell competition, it is also likely that these factors have other important targets; an observation which is supported by the fact that *Myc*-overexpressing cells do not overcome organ size control mechanisms, whereas *Yki*-overexpressing cells demonstrate unconstrained overgrowth [4,37,[50], [51], [52]].

### 2.5 NF-kB

The NF-κB transcription factors play a crucial role in the activation of genes involved in cell survival and immunity, such as growth factors and pro-inflammatory cytokines. In both *Drosophila* and mammals, signalling from Toll-like receptors leads to NF-κB nuclear localisation and transcription of target genes [53]. Intriguingly, a role for Toll receptor signalling and subsequent NF-κB activation in both *Minute* and *Myc*-induced models of cell competition has been suggested [54]. In competition induced by Myc overexpression, loser cell elimination was suppressed in a background mutant for either the *Drosophila* NF-κB homologue, Rel, or for Dredd, the caspase involved in Rel cleavage and activation. Increased Dredd activity in loser cells specifically in competition was found to result in increased Rel-mediated transcription of the pro-apoptotic protein, Hid, which was critical for their elimination. Interestingly, this study also investigated *Minute*-induced competition and found that the NF-κB homologues, Dif and Dorsal, were required for loser cell elimination in this context through their transcriptional regulation of the pro-apoptotic protein, Reaper. Thus, NF-κB-mediated upregulation of pro-apoptotic genes was required for both forms of cell competition, although each involved distinct NF-κB homologues and pro-apoptotic targets. More recently, another report confirmed the requirement for NF-κB activation in *Minute* and *Myc*-induced loser cell elimination in *Drosophila* and found that inhibition of NF-κB alone was sufficient to confer a growth advantage. However, if the flies were grown under pathogen-free conditions, inhibition of NF-κB activation no longer rescued loser cell elimination in either context [55], presumably because all the cells in the tissue have low NF-κB activity under these conditions. This observation therefore suggests that relative levels of Toll pathway signalling, and thus NF-κB activation, are critical for determining the outcome of competitive interactions.

Intriguingly, Toll signalling has also been implicated in *scrib*-induced competition. However, in contrast to *Minute* and *Myc*-mediated competition, where activation of Toll signalling and NF-κB activity was critical for loser cell elimination, in this context activated Toll signalling blocked competition and loser cell elimination [56]. Here, forced Toll signalling was sufficient to convert loser *scrib^−^* cells into super-competitors through activation of Yki. Thus, it appears that NF-κB acts in a context-dependent manner during cell competition, with pro-apoptotic targets in some cell types and pro-proliferation targets in others. Understanding what determines these differential outcomes will be an important avenue of research in the future. It should also be noted that differences exist between mammalian Toll-like receptors, which directly recognise microbial-derived products, and *Drosophila* Toll receptors, which respond to pathogens indirectly by recognising Spätzle, which is proteolytically processed in response to microbe recognition by distinct circulating receptors [53]. It is therefore unclear whether a similar mechanism could underlie fitness recognition during mammalian cell competition.

## 3 Metabolic regulation of cell competition

**Fig. 2:**
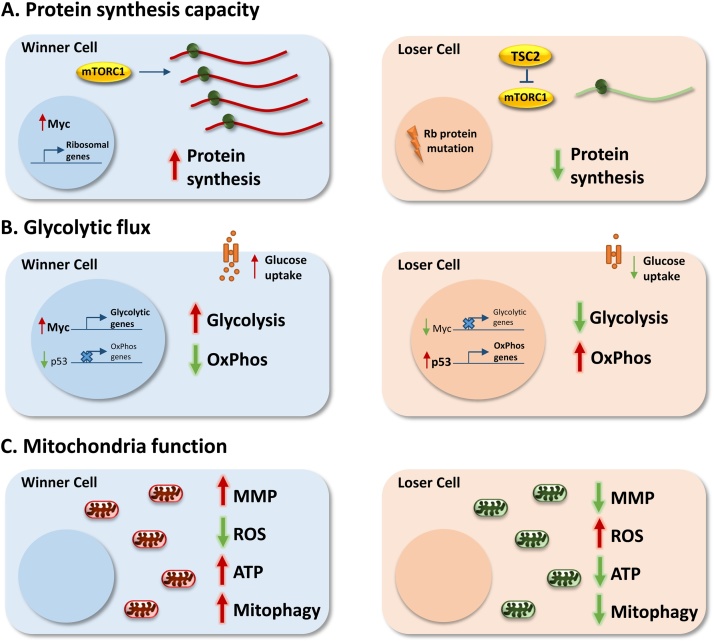
Metabolic determinants of cell fitness in competition. A. Protein synthesis capacity. Winner cells display increased protein synthesis rates compared to loser cells. This may be due to increased Myc expression or higher mTOR pathway activity in winner cells, or to ribosomal protein mutations in loser cells. B. Glycolytic Flux. Winner cells display increased glycolytic flux relative to loser cells. This may be driven by transcriptional regulation of metabolic genes by transcription factors such as Myc and p53. Glucose availability in the microenvironment may also affect glycolysis, with winner cells displaying increased uptake of glucose. C. Mitochondria Function. Loser cells may display impaired mitochondria function, resulting in decreased mitochondrial membrane potential (MMP), ATP production and mitophagy. Reactive oxygen species (ROS) production may also be elevated in loser cells with impaired mitochondrial function.

Over the last decade there has been increased evidence for the importance of metabolic changes in cell competition. In many cases this evidence has arisen from the observation that many of the transcription factors implicated in competition (discussed above) play a prominent role in regulating one or more metabolic pathways. Here we will review what is known about these pathways, with special emphasis on three that particularly stand out as likely regulators of competitive cell fitness: the rates of protein synthesis, glycolysis and mitochondrial activity (summarised in Fig. 2).

### 3.1 Rates of protein synthesis

As indicated above, the first characterised model of cell competition was impaired protein synthesis capacity relative to surrounding cells. In *Drosophila*, haploinsufficiency for multiple ribosomal protein genes results in a common ‘*Minute*’ phenotype, characterised by a reduction in the size of surface bristles and a developmental delay associated with reduced protein synthesis and proliferation rate [57]. However, despite these defects, heterozygous flies survive to adulthood and eventually reach a normal body size. In contrast, *Minute^+/−^* cell clones are eliminated by apoptosis in the imaginal wing disc in a wild-type background and fail to contribute to the adult wing [[58], [59], [60]]. Ribosomal mutations have also been shown to induce competition in mammals, as mice carrying a heterozygous mutation in the ribosomal protein *L24* gene are viable, but heterozygous clones are outcompeted in a wild-type background [61]. These results suggest that impaired protein translation is a conserved mechanism of inducing a lower cell-fitness phenotype.

Further evidence for the importance of protein synthesis rates for cell competition comes from the observation that MYC can promote protein synthesis and ribosome biogenesis through its transcriptional regulation of ribosomal proteins, ribosomal DNA, and initiation factors of translation [62]. In *Drosophila,* cells overexpressing *Myc* are no longer able to eliminate surrounding cells with lower Myc levels and are themselves eliminated if they carry the *Minute* mutation described above, which reduces ribosomal protein levels [3]. Interestingly, MYC has also been shown to regulate protein synthesis during cell competition in the early embryo [16]. Thus, both ribosomal mutations and lower MYC levels would potentially reduce the protein synthesis capacity of a cell. Furthermore, in mouse embryonic stem cells, mutation of *Tsc2*, a negative regulator of mTORC1 signalling, is sufficient to turn cells into super-competitors [26]. Given that regulation of protein synthesis is one of the most important roles of mTORC1, these results also support the possibility that relative protein synthesis levels could be an important cell-fitness determinant.

### 3.2 Rate of glycolysis

In addition to a potential role for protein synthesis in cell competition, glycolysis has also emerged as a possible mediator of cell fitness. This is in large part due to Myc, which has a well-characterised role in the promotion of aerobic glycolysis [63]. In *Drosophila* imaginal discs and S2 cells, *Myc* overexpression stimulates glucose uptake and glycolysis, and in S2 cells this was shown to be via increased expression of glycolytic genes [28]. Interestingly, this increase in glycolytic flux was accompanied by decreased mitochondrial respiration, strengthening the case that a switch to aerobic glycolysis is occurring in these cells. However, it is worth noting that in *Myc*-overexpressing S2 and imaginal disc cells, mitochondrial activity is not completely suppressed. Instead, an increase in *p53* expression occurs in these cells that promotes oxidative phosphorylation to balance the effects of *Myc* overexpression. Interestingly, in a competitive environment with wild-type cells, this metabolic flux is accentuated and *Myc*-overexpressing cells further increase their glycolysis levels in a p53-dependent manner. Importantly, the enhanced metabolism of *Myc*-overexpressing cells in a competitive environment is required for the expansion of these cells via the elimination of wild-type cells [28], providing compelling evidence for the role of glycolytic flux in cell competition.

A second instance where a shift to aerobic glycolysis affects the outcome of cell competition can be found in the apical extrusion of RasV12-transformed Madin-Darby canine kidney cells (MDCK) cells. During this process, these cells show enhanced glucose uptake, increased lactate dehydrogenase (LDHA) expression and increased lactate secretion, which is accompanied by a loss of mitochondrial activity. This phenotype was not due to a change in mitochondrial mass or number, but rather due to decreased mitochondrial function, as it could be rescued by inhibition of pyruvate dehydrogenase kinase 4 (PDK4), which diverts pyruvate from the Krebs cycle to the production of lactate [64]. Furthermore, this inhibition of PDK4 was sufficient to rescue RasV12 extrusion, highlighting the functional importance of these metabolic changes.

As mentioned above, recent studies have suggested that competitive interactions may be part of the complex interactions occurring between the tumour and stroma or even within the tumour. Tumour cells frequently demonstrate altered metabolism relative to surrounding stromal cells. In particular, higher glycolytic activity is a common feature of many tumours and this can lead to both a depletion of glucose in the microenvironment and to increased lactate release from tumour cells, which acidifies the micro-environment. Additionally, other glycolytic metabolites may also be released, which, along with lactate, are increasingly recognised to have important roles as signalling molecules. The tumour microenvironment is therefore extensively remodelled and there is evidence that this detrimentally affects the proliferation and responsiveness of surrounding stromal and immune cells [65,66].

A well-characterised example of this phenomenon is the effect of lactate on the ability of T and NK cells to mount an anti-tumoural response [65,66]. Lactate accumulation was shown to inhibit T and NK cell activation and infiltration of the tumour in melanomas [67]. One potential mechanism for this could be that lactate export by activated T cells, which themselves have high levels of glycolysis, is inhibited by the presence of high extracellular levels of lactate, thus disturbing their metabolism and function [68]. Additionally, lactate also affects myeloid cell activation, suppressing monocyte activation and dendritic cell differentiation [69,70]. This lactate-induced immune suppression likely has an impact on tumour progression as a correlation can be observed between the concentration of lactate in the tumour and the incidence of metastases and recurrence in cervical cancers [71].

Glucose availability in the tumour micro-environment has also been shown to impact on the responsiveness of infiltrating T cells. In renal carcinoma higher expression of the glucose uptake channel, GLUT-1, was found to correlate with lower CD8^+^ T cell infiltration of the tumour [72]. Intra-tumoural CD4^+^ and CD8^+^ T cells also demonstrate decreased IFNγ production and effector function as a result of glucose restriction in the tumour microenvironment [73,74]. Boosting tumour cell glycolysis by overexpressing MYC, glycolytic enzymes or GLUT-1 increased tumour progression, while suppression of glycolysis by checkpoint blockade therapy restored T cell responses and resulted in tumour regression [73]. Inhibiting glycolysis or targeting glucose uptake channels in tumours could therefore be a potential therapeutic strategy as it would increase the glucose available to circulating immune cells, thus enhancing their function. Additionally, in human glioblastoma samples, the cancer stem cell (CSC) population exhibits an increased efficiency of glucose uptake relative to non-CSCs and this gives them a selective advantage under low glucose conditions [75]. Furthermore, targeting the glucose uptake channel responsible for this advantage reduces the tumourigenic potential and growth of these CSCs. Therefore, nutrient competition both within tumours and between tumour and stromal cells or immune cells can influence tumour progression through mechanisms akin to cell competition.

### 3.3 Mitochondrial function

Mitochondria are classically seen as the powerhouses of cells, where generation of ATP occurs by oxidative phosphorylation (OXPHOS). Significantly, in addition to supplying energy to cells, mitochondria are also involved in many other cellular processes, such as calcium and reactive oxygen species (ROS) signalling, apoptosis, the immune response and the cell stress response [[76], [77], [78], [79], [80]]. Mitochondrial function was already suggested to have a role in cell-cell communication in *Drosophila* imaginal discs as mitochondrial defects in Ras-activated epithelial cells lead to oxidative stress that promotes non-cell autonomous growth of surrounding tissue [81,82]. Recently, mitochondria function has also been implicated in cell competition. For example, analysis of the *Minute* and *Mahjong* mutations eliminated by competition in the *Drosophila* wing disc found that both had a common signature of oxidative stress. Mutant cell survival was dependent on the activity of Nrf2, a transcription factor central to the oxidative stress response. The authors observed that, while endogenous Nrf2 expression is required for loser survival in a homogeneous environment, in a competitive context Nrf2 overactivation promotes their elimination [35]. Given that the mitochondria are considered to be the major origin of ROS, these results raise the possibility that defective mitochondrial function is underlying the elimination of *Minute* and *Mahjong* mutant cells.

Interestingly, *Drosophila Scribble knock-down (scrib^KD^*) cells, that are eliminated by cell competition, also display a cell-intrinsic signature of oxidative stress and mitochondrial dysfunction. In the *Drosophila* wing imaginal discs *scrib^KD^* cells cause overgrowths and show a loss of mitochondria membrane potential, reduced ATP production, increased ROS and impaired mitophagy (the mechanism of clearing defective mitochondria). Furthermore, the authors found that promoting mitophagy rescues the wing distortion and reduces the death rates of these mutant larvae by half, supporting hypothesis that mitochondria dysfunction plays a role in the dysregulated growth of these cells [83].

Further evidence for the role of mitochondrial activity in determining cell fitness comes from the analysis of the behaviour of immortalized mammalian cell lines. Here, characterisation of stochastically arising sub-clones of human (U2OS), mouse (3T3) and canine (MDCK) cell lines found that the proliferation rates and saturation densities of each cell line predicted their competitive ability, with the faster proliferating cells being invariably the winners. Furthermore, the authors found that the competition between these cells did not require *de novo* RNA synthesis, but rather was abolished under hypoxia or limiting glutamine concentrations. Both these factors have a direct role in determining OXPHOS output and the observation that dissipating the proton gradient of the electron transport chain with CCCP gave a dose-dependent rescue of loser cell elimination, further supports the possibility that mitochondrial output was a key determinant of cell competition in these cells [84]. Therefore, taken together, all the above studies suggest an important role of mitochondrial performance in the origin of competitive interactions.

## 4 Conclusion and future perspectives

Understanding the parameters that determine relative cell fitness during cell competition is important as it provides an avenue for the manipulation of this process. Given the plethora of roles that cell competition has been postulated to play, from the optimisation of tissue fitness during ageing [85], to tumour promotion as well as tumour suppression [5], this manipulation has the potential to promote either cell replacement during tissue regeneration or to prevent the expansion of cancer cells. As reviewed above, a number of transcriptional regulators have been shown to profoundly affect the competitive ability of the cell. However, one recurrent theme is that the precise mechanism of action of these transcription factors appears to be highly context dependent and no obvious target for their activity has been identified during cell competition. One interesting possibility that has been put forward is that hypertranscription, or the ability of a cell to globally upregulate their transcriptome during specific transitions, may be important for cell competition [86]. Both MYC and YAP/TAZ can regulate hypertranscription (reviewed in [86]), therefore it is possible that this may provide cells with a competitive advantage over their neighbours. But how cells can measure their relative global transcriptional output is a question that needs resolving.

An alternative possibility that we have explored here is that an important mode by which these transcription factors act during cell competition is through the regulation of one or more metabolic pathways. For example, the mechanism by which Myc confers a competitive cell advantage appears to be through both increased rates of glycolysis as well as via the regulation of protein synthesis. Given the complexity and inter-relatedness of the different cellular metabolic pathways, this raises the intriguing possibility that the metabolic state of the cell may determine its competitive fitness. Following this argument, it is possible that cells may measure their relative fitness levels through the exchange of metabolites. Again, untangling if this is the case and how relative levels of metabolites can be measured between cells is a challenge that will likely fuel the growing cell competition field for years to come.

## Declaration of Competing Interest

We have no competing interests.
